# Theabrownin Inhibits Cell Cycle Progression and Tumor Growth of Lung Carcinoma through c-myc-Related Mechanism

**DOI:** 10.3389/fphar.2017.00075

**Published:** 2017-02-27

**Authors:** Li Zhou, Feifei Wu, Wangdong Jin, Bo Yan, Xin Chen, Yingfei He, Weiji Yang, Wenlin Du, Qiang Zhang, Yonghua Guo, Qiang Yuan, Xiaoqiao Dong, Wenhua Yu, Jin Zhang, Luwei Xiao, Peijian Tong, Letian Shan, Thomas Efferth

**Affiliations:** ^1^Institute of Orthopaedics and Traumatology, Zhejiang Chinese Medical UniversityHangzhou, China; ^2^Institute for Cell-Based Drug Development of Zhejiang Province, S-Evans Biosciences Inc.Hangzhou, China; ^3^The Second Clinical Medical College, Zhejiang Chinese Medical UniversityHangzhou, China; ^4^Hangzhou First People’s HospitalHangzhou, China; ^5^Theabio Co., LtdHangzhou, China; ^6^Department of Pharmaceutical Biology, Institute of Pharmacy and Biochemistry, Johannes Gutenberg University of MainzMainz, Germany

**Keywords:** theabrownin, lung cancer, TUNEL, cell cycle, c-myc

## Abstract

Green tea, the fresh leaves of *Camellia sinensis*, is not only a health-promoting beverage but also a traditional Chinese medicine used for prevention or treatment of cancer, such as lung cancer. Theabrownin (TB) is the main fraction responsible for the medicinal effects of green tea, but whether it possesses anti-cancer effect is unknown yet. This study aimed to determine the *in vitro* and *in vivo* anti-lung cancer effect of TB and explore the underlying molecular mechanism, by using A549 cell line and Lewis lung carcinoma-bearing mice. In cellular experiment, MTT assay was performed to evaluate the inhibitory effect and IC50 values of TB, and flow cytometry was conducted to analyze the cell cycle progression affected by TB. In animal experiment, mice body mass, tumor incidence, tumor size and tumor weight were measured, and histopathological analysis on tumor was performed with Transferase dUTP nick-end labeling staining. Real time PCR and western blot assays were adopted to detect the expression of *C-MYC* associated genes and proteins for mechanism clarification. TB was found to inhibit A549 cell viability in a dose- and time-dependent manner and block A549 cell cycle at G0/G1 phase. Down-regulation of c-myc, cyclin A, cyclin D, cdk2, cdk4, proliferation of cell nuclear antigen and up-regulation of p21, p27, and phosphate and tension homolog in both gene and protein levels were observed with TB treatment. A c-myc-related mechanism was thereby proposed, since c-myc could transcriptionally regulate all other genes in its downstream region for G1/S transitions of cell cycle and proliferation of cancer cells. This is the first report regarding the anti-NSCLC effect and the underlying mechanism of TB on cell cycle progression and proliferation of A549 cells. The *in vivo* data verified the *in vitro* result that TB could significantly inhibit the lung cancer growth in mice and induce apoptosis on tumors in a dose-dependent manner. It provides a promising candidate of natural products for lung cancer therapy and new development of anti-cancer agent.

## Introduction

Lung cancer has the highest incidence rate of cancer and becomes the leading cause of cancer death (about 1.38 million deaths annually) in the world (Siegel et al., 2015; Torre et al., 2015). The estimated new cases and deaths from lung cancer in the United States in 2016 were 224,390 and 158,080, respectively (American Cancer Society, 2016). Non-small cell lung cancer (NSCLC) comprises 80∼85% of lung cancer cases, with an extremely poor 5-year survival rate of <15% and only 5∼10% survival rate for advanced NSCLC (Pore et al., 2013). Despite considerable advances in lung cancer therapy, including chemotherapy, radiotherapy, and immunotherapy, the overall success in treating NSCLC remains unsatisfactory due to the limited efficacy and/or serious side effects (Niyazi et al., 2011; Iwamoto, 2013). Therefore, much attention has been focused on anti-cancer agents derived from herbal medicines to change the unsatisfactory situation, owing to their extensive efficacy and low toxicity.

Green tea, the fresh leaves from the evergreen plant *Camellia sinensis* (L.) O. Kuntze (Theaceae), is the most ancient health-promoting beverage in the world. It has been officially described as a medicine by the earliest national pharmacopeia ‘*Xin Xiu Ben Cao’* (Newly Revised Materia Medica, AD 659) in Tang Dynasty of China, and was characterized with bitter and sweet flavor as well as cold and non-toxic nature, which can function to eliminate the heat, phlegm, and toxins from body. In the traditional Chinese medicine (TCM) theory, heat, phlegm and toxins are considered as causes of many chronic diseases, such as cancer, resulting in the application of green tea by TCM for the prevention/treatment of cancer (Yang et al., 2014). In recent years, the anti-cancer activity of green tea, especially against lung cancer, has been evidenced by various studies (Imai et al., 1997; Yang et al., 2000; Cabrera et al., 2006; Suqanuma et al., 2011). As the main pigments of green tea, theabrownin (TB), theaflavin (TF) and thearubigin (TR) together determine the color, taste, and the bioactivity of the tea liquor (Roberts et al., 1957). Of these pigments, TB is a major fraction governing the medicinal effects of green tea, such as cholesterol-lowering effect in relieving fatigue and reducing blood lipid levels (Gong et al., 2010). In view of the TB’s key role in green tea, it can be expected that TB has a certain anti-lung cancer potential representative for the same activity of green tea. However, whether it does is unknown yet.

Cell proliferation is dependent on the progression of the cell cycle which is composed of the G_1_, S, G_2_ and M phases, and the transition from the G_1_ to S phase is critical as it controls the subsequent progress of the cell cycle (Luo et al., 2016). The G_1_ to S phase transition is tightly regulated by the activation of CDKs, which act consecutively in G_1_ to initiate the S phase and in the G_2_ phase to initiate mitosis (Genovese et al., 2006; Lim and Kaldis, 2013). Previous studies have reported that many natural products could inhibit cell cycle progression (G_0_/G_1_ arrest) and proliferation of the NSCLC cells (A549 cell line), indicating G_0_/G_1_ checkpoint in cell cycle as one of the most conspicuous targets for anti-cancer agents. For determining the anti-lung cancer effect of TB, this study employed A549 cells and conducted cellular and molecular assays to evaluate TB’s effect on cell cycle progression and proliferation of the NSCLC cells and explore the underlying mechanism.

## Materials and Methods

### Chemicals and Materials

Theabrownin powders (>90% of purity) and green tea crude extract were provided by Theabio Co., Ltd (Hangzhou, China; Batch number: 20151105001). Roswell Park Memorial Institute (RPMI) 1640 medium, fetal bovine serum (FBS), and 0.25% trypsin were obtained from Gibco BRL (Grand Island, NY, USA). 3-(4,5-dimethylthiazol-2-yl)-2,5-diphenyltetrazolium bromide (MTT) and dimethyl sulfoxide (DMSO) were obtained from Sigma (St. Louis, MO, USA). Cell cycle kit was obtained from BD Biosciences (San Jose, CA, USA). All antibodies were purchased from Cell Signaling Technology (CST, Danvers, MA, USA). Trizol reagent and real time polymerase chain reaction (real time PCR) kit were purchased from TaKaRa (Dalian, China). Transferase dUTP nick-end labeling (TUNEL) staining kit was purchased from Roche Applied Science (Indianapolis, IN, USA).

### Cell Line and Animal

Human NSCLC A549 cell line and mouse Lewis lung carcinoma (LLC) cell line were obtained from Shanghai Cell Bank of Chinese Academy of Sciences (Shanghai, China) and cultured in RPMI-1640 medium containing 10% FBS at 37°C in a humidified 5% CO_2_ incubator. The medium was changed daily and the cells were used in their logarithmic growth phase. Male C57BL/6 mice weighing 20∼22 g were purchased from Shanghai Laboratory Animal Center of Chinese Academy of Sciences (Grade SPF II, SCXK2012-0002) and housed under controlled pathogen-free conditions with a 12 h light/dark cycle and allowed food and water *ad libitum*. All animals were treated in strict accordance with the China legislation on the use and care of laboratory animals. The animal experiment was approved by the Medical Norms and Ethics Committee of Zhejiang Chinese Medical University.

### MTT Assay

MTT assay was performed to evaluate the cell viability of A549 cells, as described in our previous report (Wu et al., 2016). Cells were seeded on 96-well plates with density of 5 × 10^3^ cells/well in 200 μl medium for 24 h and then treated with TB at different concentrations (0, 5, 25, 50, 100, 150, 200, 300 μg/ml) for 24, 48, and 72 h. Each 20 μl MTT solution (5.0 mg/ml) was added to each well and incubated at 37°C for 4 h. Then 150 μl DMSO was added in each well to dissolve the MTT formazan crystals and the optical density value (OD value) was measured at 490 nm with a microplate reader (Bio-Rad, Berkeley, CA, USA). Inhibitory rate (%) = [1- (TB - treated OD / untreated OD)] × 100%. The 50% inhibitory concentrations (IC_50_) for 24, 48, and 72 h were calculated by regression analysis, respectively.

### Cell Cycle Analysis

Cell cycle analysis was performed with flow cytometry, as described in our previous report (Wu et al., 2016). Briefly, A549 cells were seeded on 6-well plates with density of 3 × 10^5^ cells/well for 24 h, followed by TB treatment for 24, 48, and 72 h. The cells were harvested and washed with PBS thrice and suspended in cold phosphate-buffered saline. Cells were then stained with PI/RNase staining solution (20 μg/ml PI, 10 μg/ml DNase free RNase) at 37°C in dark for 30 min. The cell cycle was analyzed in three replicates using flow cytometry (Beckman Coulter, USA).

### Real Time PCR Analysis

After TB treatment, gene expression in A549 cells was detected by real time PCR assay on an ABI QuantStudio^TM^ 7 Flex Real-Time PCR System (Applied Biosystems, Foster City, CA, USA), according to our previous report (Wu et al., 2016). The total RNA of the cells in each group was extracted using Trizol reagent and synthesized to cDNA via reverse transcription. PCR reaction system had a 20.0 μl volume: 10.0 μl SYBR^®^ Premix Ex Taq II (Tli RnaseH Plus), 0.8 μl PCR Forward Primer, 0.8 μl PCR Reverse Primer, 2.0 μl template cDNA, 0.4 μl ROX Reference Dye, and 6.0 μl ddH_2_O. The qPCR reaction condition was set to 95°C for 30 s initial denaturation, 40 cycles of 95°C for 5 s denaturation, 60°C for 34 s annealing, and 72°C for 40 s extension. At the end of each reaction, a melting curve analysis was performed. β-actin was used as the reference gene and 2^-ΔΔCT^ method was applied to analyze the relative expression of gene *C-MYC* (forward: GCCACGTCTCCACACATCAG; backward: TGGTGCATTTTCGGTTGTTG), *CYCLIN D* (forward: CAATGACCCCGCACGATTTC; backward: CATGGAGGGCGGATTGGAA), *CYCLIN A* (forward: GGATGGTAGTTTTGAGTCACCAC; backward: CACGAGGATAGCTCTCATACTGT), *P21* (forward: GGCAGACCAGCATGACAGATT; backward: GCGGATTAGGGCTTCCTCT), *P27* (forward: TGGAGAAGCACTGCAGAGA; backward: GCGTGTCCTCAGAGTTAGCC), *CDK2* (forward: GCTAGCAGACTTTGGACTAGCCA; backward: AGCTCGGTACCACAGGGTCA), *CDK4* (forward: AAATCTTTGACCTGATTGGG; backward: CCTTATGTAGATAAGAGTGCTG), *PCNA* (forward: GTAGTAAAGATGCCTTCTGGTG; backward: TCTCTATGGTAACAGCTTCCTC), and *PTEN* (forward: CCGAAAGGTTTTGCTACCATTCT; backward: AAAATTATTTCCTTTCTGAGCATTCC).

### Western Blot Analysis

Following TB treatment, A549 cells were harvested and washed thrice with cold PBS. As previously described (Wu et al., 2016), the cell pellets were resuspended in cell lysis buffer (50 mM Tris-HCl pH 7.4, 150 mM NaCl, 1 mM EDTA, 1% Triton, 0.1% SDS, 5 μg/ml leupeptin, and 1 mM PMSF) for 30 min on ice repeated freezing and thawing for three times. After cold centrifugation at 14,000 r/min for 20 min, the supernatant was collected and stored at -80°C. The lysed protein was quantified using a BCA protein assay kit. Appropriately 20 μg of lysed protein was boiled in 4x sample buffer and separated by a denaturing SDS-PAGE (8∼12%). The gels were transferred onto polyvinylidene fluoride membranes (Millipore, Billerica, MA, USA) and blocked with 5% non-fat milk for 2 h, followed by overnight incubation at 4°C with the primary antibodies: c-Myc, Cyclin A, Cyclin D, P21, P27, Cdk2, Cdk4, proliferation of cell nuclear antigen (PCNA), phosphate and tension homolog (PTEN), and β-tubulin. Following incubation with peroxidase-conjugated goat anti-rabbit IgG at room temperature for 2 h, proteins were visualized using enhanced chemiluminescence kit (Amersham Pharmacia Biotech, Little Chalfont, UK) and detected using a chemiluminescence analyzer.

### Lewis Lung Carcinoma (LLC)-Bearing Mouse Model Establishment and TB Treatment

To establish the lung cancer model of mouse, LLC cells (1.0 × 10^7^ cells in 0.1 ml of PBS) were injected subcutaneously into 40 mice. The mice were randomly divided into four groups of 10 mice each: model group, low-dose TB group, middle-dose TB group, and high-dose TB group. After the injection, mice in the low-dose TB, middle-dose TB, and high-dose TB groups were daily treated with 0.1 g/kg, 0.2 g/kg, and 0.4 g/kg mice body weight by oral administration for 21 days, respectively.

### Evaluation of *In vivo* Anti-tumor Effect of TB

Mouse body weight of each mouse in each group was measured per 3 days, and the tumor growth was observed for recording tumor incidence. On day 21, all mice were sacrificed and each tumor was sampled for size and weight measurement. The tumor volume was estimated by the formula (Gu et al., 2016):

$$V({mm}^{3})=\frac{\left({length\times width}^{2}\right)}{2}.$$

### *In situ* Apoptosis Assay

As previously described, the tumor tissue in each mouse was dissected, fixed in 10% formaldehyde for 24 h, and embedded in paraffin wax. Sections (2∼3 μm) were stained by terminal deoxynucleotidyl TUNEL using the *in situ* cell death detection kit (Roche) according to the manufacturer’s instructions to detect the apoptotic cells (Zhang et al., 2017). Apoptotic cells were manifested by green staining in the nuclei under microscopy and five microscopic fields (×400) of each section were randomly examined, and the positive cells were counted by a single-blinded observer in a coded randomized order.

### Statistical Analysis

Data were expressed as mean ± SD and subjected to one-way ANOVA, followed by Fisher’s least significant difference (LSD) comparison. All analyses were performed using an updated version of DPS software (Tang and Feng, 2007).

## Results

### TB Inhibits Cell Viability of A549 Cells

**FIGURE 1:**
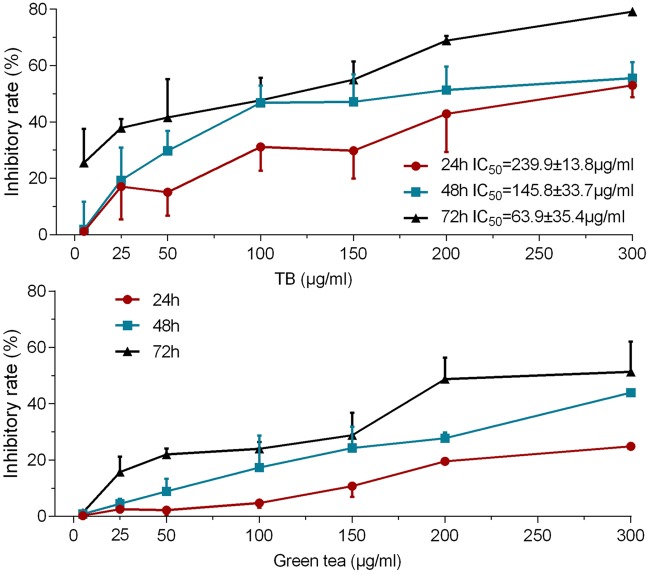
**Effect of Theabrownin (TB) and green tea extract on cell viability of A549 cells determined by MTT assay.** Values were presented as mean ± SD (*n* = 5).

As shown by MTT assay in **Figure 1**, TB treatment caused an obvious inhibition of A549 cell viability at various concentrations (5∼300 μg/ml) for 24, 48, and 72 h. The inhibitory effect was increased with increasing concentrations at each time point and was also increased with increasing time period at each concentration point, indicating a dose- and time-dependent manner of TB. The IC_50_ values were 239.89 ± 13.78, 145.80 ± 33.67, and 63.93 ± 35.44 μg/ml for 24, 48, and 72 h treatment, respectively. The green tea extract could also induce cell viability inhibition at the same concentration range for 24, 48, and 72 h, but the effect was weaker than that of TB. The 200 μg/ml was selected as a high effective dose of TB for the following assays.

### TB Induces Cell Cycle Arrest at G_0_/G_1_ Phase in A549 Cells

**FIGURE 2:**
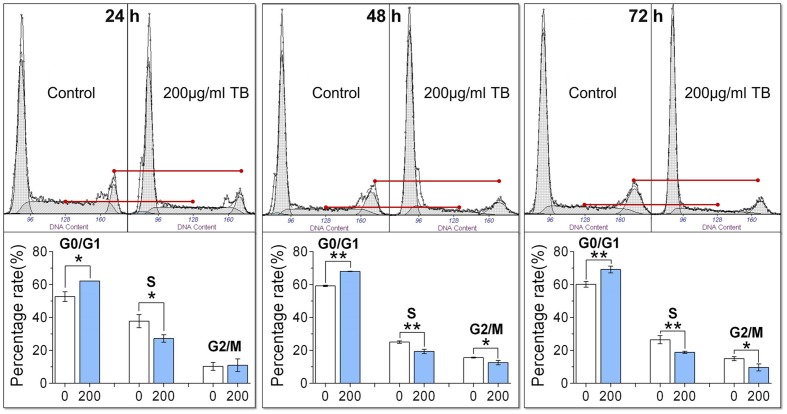
**Effect of TB on cell cycle of A549 cells determined by flow cytometry.** Values were presented as mean ± SD (*n* = 3). ^∗^*P* < 0.05 and ^∗∗^*P* < 0.01 for comparison between control group and TB group.

As shown by flow cytometry in **Figure 2**, TB-treated A549 cells was accumulated in the G_0_/G_1_ phase of the cell cycle, with a significant increase of cell number in the G_0_/G_1_ phase as compared to controls (all *P* < 0.05). Correspondingly, the number of cells in the S phase and G_2_/M phase were significantly decreased except for that in the G_2_/M phase at 24 h. These trends indicated that TB could induce cell cycle arrest of A549 cells in G_0_/G_1_ phase.

### TB Modulates Cell Cycle Associated mRNA and Protein Expression

**FIGURE 3:**
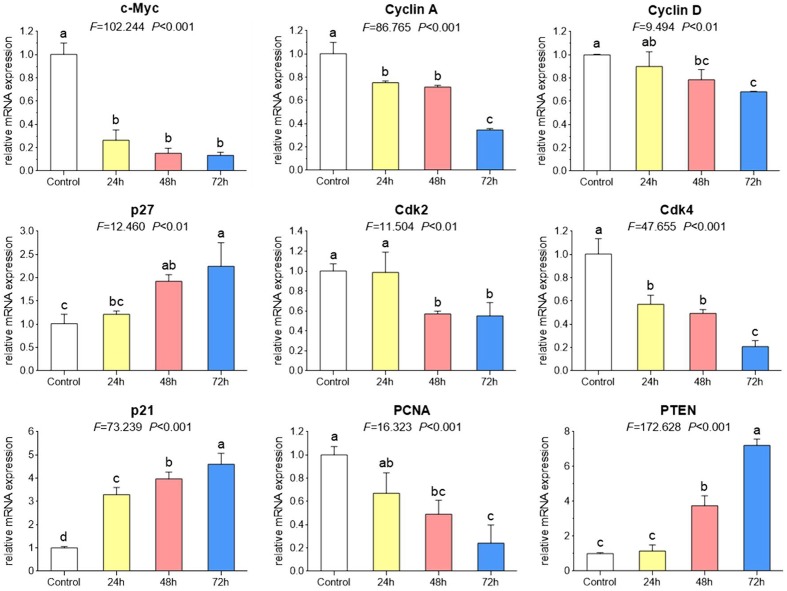
**Relative mRNA expression of target genes in A549 cells with TB treatment at 200 μg/ml for 24, 48, and 72 h.** Values are presented as mean ± SD of three replicates. Different symbols (a, ab, b, c, bc, d) indicate significant difference between groups [Fisher’s least significant difference (LSD), *P* < 0.05], and the values decrease with the order from a to d.

**FIGURE 4:**
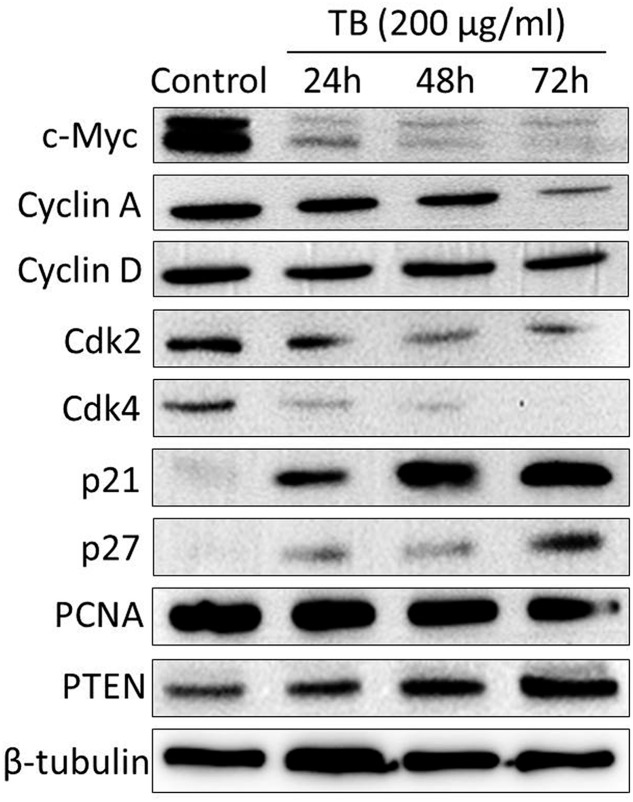
**Protein expression of target genes in A549 cells with TB treatment at 200 μg/ml for 24, 48, and 72 h**.

As shown by real time PCR in **Figure 3**, the mRNA expression of cell cycle associated genes were significantly modulated by TB (200 μg/ml) after 24 to 72 h treatment. Of these genes, *C-MYC. CYCLIN A. CYCLIN D. CDK2. CDK4*, and *PCNA* were down-regulated while *P21. P27*, and *PTEN* were up-regulated, as compared to the control. Mostly, the modulation effect of TB on those genes displayed a time-dependent manner. In accordance with this result, western blot analysis also showed a similar trend of protein expressions encoded by those genes (**Figure 4**).

### *In vivo* Anti-tumor Effect of TB

**FIGURE 5:**
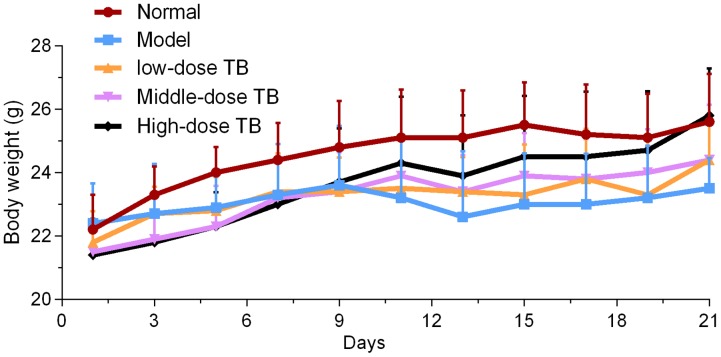
**Mice body weight of all groups (*n* = 10)**.

**FIGURE 6:**
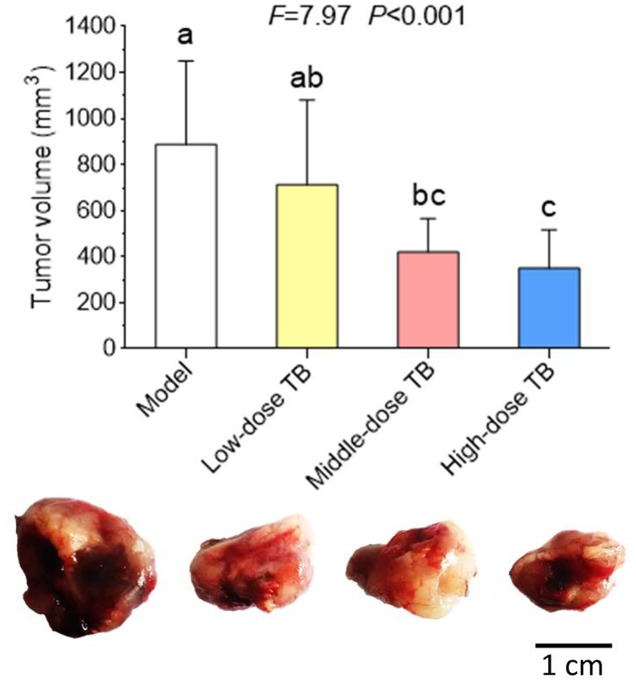
**Tumor volume of mice in all groups (*n* = 10).** Different symbols (a, ab, bc, c) indicate significant difference between groups (Fisher’s LSD, *P* < 0.05), and the values decrease with the order from a to c. Bar = 1 cm.

**FIGURE 7:**
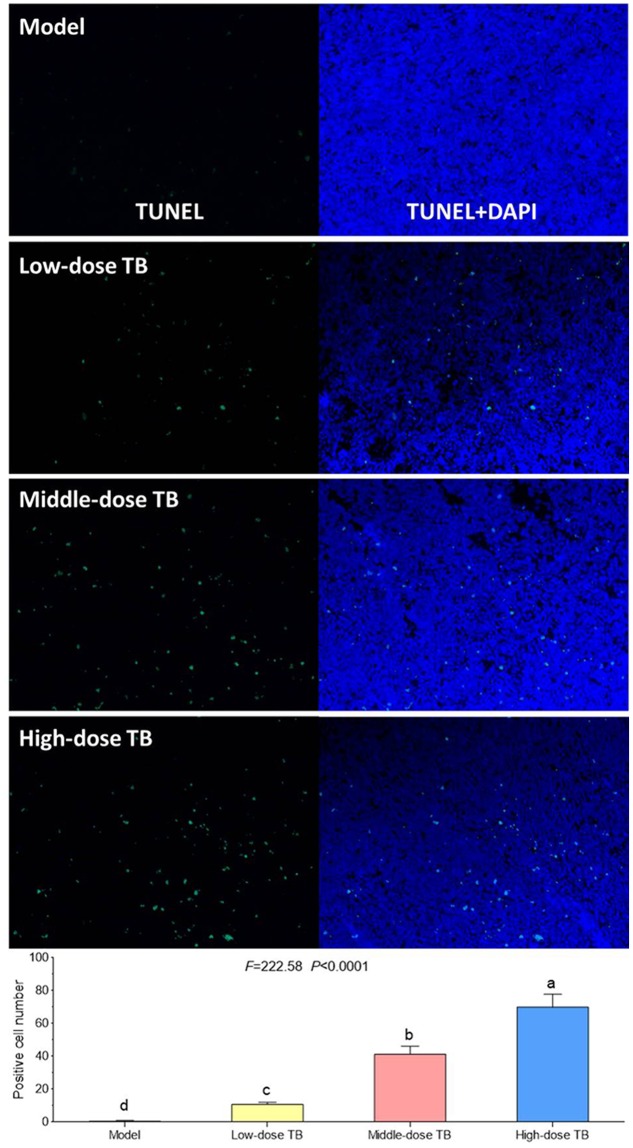
**Transferase dUTP nick-end labeling (TUNEL) and DAPI staining on mice tumors and the TUNEL-positive cell number comparison among all groups.** Different symbols (a, b, c, d) indicate significant difference between groups (Fisher’s LSD, *P* < 0.05), and the values decrease with the order from a to d.

As shown in **Figure 5**, the mice body weight in LLC model group was gradually changed after LLC injection and even decreased after day 9. In TB-treated groups, the mice body weight kept increase with a dose dependent manner of TB treatment, suggesting a recovery of physiological state induced by TB. In each group, LLC tumor growth could be visually seen at day 9 and the tumor incidence among all groups were differed, in which the model group had the highest incidence but the TB-treated groups had lesser ones (**Table 1**). On day 21, the tumor volume in the model group was significantly bigger than that in the TB-treated groups, showing that TB could significantly inhibit the LLC growth in a dose-dependent manner (**Figure 6**). TUNEL assay also showed a consistent result that TB could induce remarkable apoptosis in LLC tumors in a dose-dependent manner (**Figure 7**).

**Table 1:** Tumor incidence (%) of Lewis lung carcinoma (LLC)-bearing mouse model with TB treatment (*n* = 10).

	Day1	Day3	Day5	Day7	Day9	Day11	Day13	Day15	Day17	Day19	Day21
Normal	0	0	0	0	0	0	0	0	0	0	0
Model	0	0	0	0	90	100	100	100	100	100	100
Low-dose TB	0	0	0	0	40	70	100	100	100	100	100
Middle-dose TB	0	0	0	0	10	50	90	100	100	100	100
High-dose TB	0	0	0	0	10	40	100	100	100	100	100


## Discussion

There has been a growing interest in ethnopharmacological use of TCMs as a new strategy for anti-cancer treatment, owing to their advantages of high bioactivities and low toxicity. As the most common beverage and also a TCM, green tea was found with anti-cancer activity against e.g., lung cancer. A 10-year epidemiological survey on 8,552 individuals has concluded that over 10 cups of green tea per day could delay the cancer onset among the general population (Nakachi et al., 2000). The need of drinking over 10 cups means that the anti-cancer effect of green tea may rely on the accumulation of some kind of low-content component. Thus, to find out what component dominates the anti-cancer effect of green tea is of practical necessity, so that it can be concentrated and developed into an efficient agent for cancer treatment. In the present study, we focused on the anti-cancer effect and the underlying mechanism of TB on NSCLC cells, in view of the fact that green tea has anti-NSCLC effect and TB acts as its main component (Roberts et al., 1957; Khan et al., 2009; Yang et al., 2009).

Our results demonstrated that TB inhibited A549 cell viability in a dose- and time-dependent manner and blocked A549 cell cycle at G_0_/G_1_ phase (**Figure 1**). The real time PCR and western blot analyses have clarified the associated mechanism that TB induced cell cycle arrest through down-regulation of c-myc, cyclin A, cyclin D, cdk2, cdk4, PCNA and up-regulation of p21, p27, and PTEN (**Figure 2**). The c-myc is a transcriptional factor and a well known oncogene expressed in G_1_ phase of cell cycle, which induces DNA synthesis by direct control of the initiation of DNA replication or by transcriptional regulation of the genes involved in DNA replication (Dominguez-Sola et al., 2007). Dysfunctional overexpression of c-myc is commonly observed in a wide range (roughly 70%) of human tumors and intensely associated with the cell proliferation in numerous malignancies, whereas inhibition of c-myc results in cell cycle arrest and decreased cell proliferation (Nesbit et al., 1999; Delmore et al., 2011). The downstream genes targeted by c-myc for cell cycle regulation are cyclin A, cyclin D, PCNA, p21, p27, PTEN, etc. (Hoang et al., 1994; Grandori et al., 2000; Guo et al., 2000; Zhang et al., 2010; Ott et al., 2013; Shao et al., 2014).

The cyclin A is a member of cyclin family, the transcription of which is tightly regulated and synchronized with cell cycle progression (Soucek et al., 1997). It promotes both cell cycle G_1_/S and G_2_/M transitions by binding and activating cdk2, a member of the cyclin-dependent kinase family of serine/threonine protein kinases (Mendoza et al., 2003). Complex of cyclin A and cdk2 is required to progress through the S phase and plays important role in G_1_ cell cycle arrest of cancer cells (Morgan, 1995; Kokontis et al., 2014). As another downstream gene of c-myc and also cyclin family member, cyclin D is the major G_1_ phase cyclin and is overexpressed in most cancer cells (Christopher et al., 2004). Complex of cyclin D and cdk4 is necessary to regulate G_1_/S transition in the cell cycle progression (Sherr, 1996). PCNA is also a c-myc-targeted gene that produces a 36 kD nuclear protein in late G_1_ and S phases for cell proliferation, DNA repair and cell cycle control (Strzalka and Ziemienowicz, 2010). Down-regulation of PCNA could induce cell cycle arrest and cell proliferation inhibition of cancer cells (A549; Chairuangkitti et al., 2013). The cdk inhibitors, p21 and p27, are known downstream targets of c-myc and capable of binding to and inhibit the active cyclin/cdk complexes as well as PCNA in the nucleus (Grandori et al., 2000). Through their binding to cdk2 and cdk4, p21 and p27 have been reported to induce blockade of G_1_/S transitions of cell cycle and then premature senescence in cancer cells (Kuilman et al., 2010). Likewise, by binding to PCNA, p21 blocks the stimulation ability of PCNA to DNA polymerases and leads to DNA replication inhibition and cell cycle arrest (Moldovan et al., 2007; Abbas and Dutta, 2009). PTEN is a crucial tumor suppressor in many cancer types, the gene of which is negatively regulated by c-myc (Zhang et al., 2010). It decreases cell proliferation through cell cycle arrest in G_0_/G_1_ phase and is frequently mutated or lost in many kinds of cancers (Gottschalk et al., 2001). The decrease of PTEN expression is correlated with poor prognosis of cancers, and vice versa (Jiang and Liu, 2008). The above are all c-myc targets associated with G_1_/S transitions of cell cycle and proliferation of cancer cells. In this study, with TB treatment, they showed a consistent expression trend with each other, toward G_1_ cell cycle arrest and proliferation inhibition of A549 cells. Therefore, a mechanism can be proposed that TB directly inhibited the overexpression of *C-MYC* and then transcriptionally regulated the downstream genes as we listed above. It has been found that MAPK and NF-κB are the upstream signaling molecules of *C-MYC*, activation of which positively correlates with c-myc-mediated cell proliferation and cell cycle progression in lung cancer (Tang et al., 2013; Lim et al., 2016). Other publications reported that tea and its components could inhibit human cell growth and migration by inhibiting MAPK or NF-κB signaling (Karki et al., 2011; Liu et al., 2016; Zhou et al., 2016), suggesting the potential importance of those signaling molecules in the role of TB. Therefore, more studies in future are needed to explore MAPK and NF-κB signaling pathways in the c-myc-related mechanism of TB.

The induction of cell cycle arrest is an important mechanism involved in anticancer agents (Swanton, 2004). In particular, cell cycle arrest in the G_1_ phase is of potentially great clinical value, because the G_1_ phase is the most sensitive time point for cancer therapy. A cell cycle restriction point occurs in the mid-G_1_ phase, and after this point, cancer cells become independent of growth factors and become committed to cell division (Kastan and Bartek, 2004). For example, cancer cells appear to be maximally sensitive to cisplatin in the G_1_ phase and minimally sensitive in peak DNA-synthesis phase, then with a twofold decrease in the S phase (Shah and Schwartz, 2001). Studies have reported that the down-regulation of c-myc expression can also enhance sensitivity to chemotherapeutic drugs as cisplatin (Xie et al., 2006, 2009). Taken together, TB may not only suppress cancers by itself but also facilitate the effect of chemotherapeutics by increasing cancer cell sensitivity, owing to its c-myc-related mechanism of G_1_ cell cycle arrest.

**FIGURE 8:**
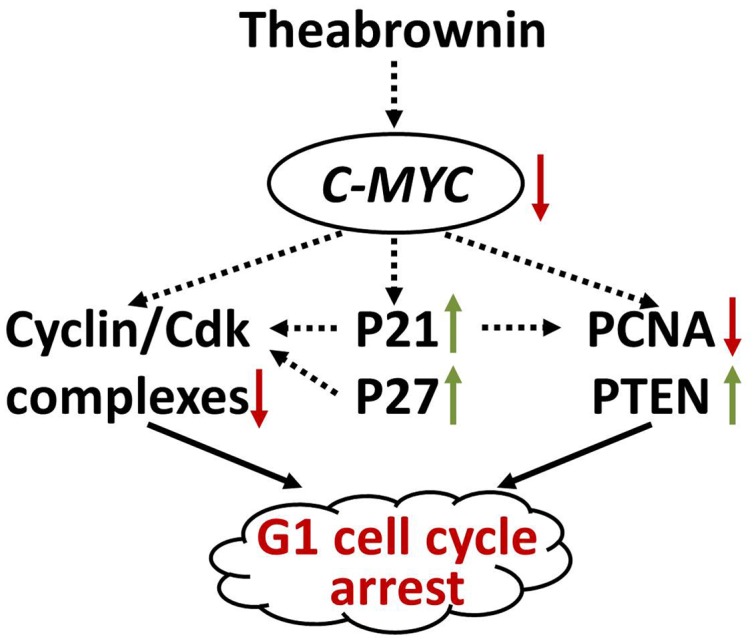
**c-myc-related mechanism of TB on A549 cells**.

In the present study, TB induced G_1_ cell cycle arrest and proliferation inhibition of A549 cells by regulation of c-myc and its downstream genes, indicating an anti-NSCLC effect through c-myc-related mechanism (**Figure 8**). The results provide evidences that TB dominates and represents the anti-cancer effect of green tea, and that it may facilitate chemotherapy by enhancing cell sensitivity of cancers. We have also evaluated the effect of TB on human embryonic lung fibroblast (HFL1 cell line) by MTT assay and found its cytotoxic dose was upon 1000 μg/ml (data not shown). Within the mentioned dose range between 5 to 300 μg/ml, no toxicity was seen in MTT assay. Therefore, TB induces specific anti-lung cancer effect in this study. Therefore, TB deserves further study in combination with chemotherapeutics for the treatment of NSCLC *in vivo*, contributing to the development of a new anti-cancer agent from green tea.

## Author Contributions

LZ, FW, and WJ performed the main experiments of this work; WYa, WD, QZ, YG, and JZ contributed to the materials acquisition and data analysis for this study; LS and QY designed this work and drafted the manuscript; LX, PT, XD, WYu, and TE improved the design and draft of this work. BY, XC, and YH conducted the animal experiment. All listed authors approved the version for publication, and agreed to be accountable for all aspects of this work.

## Conflict of Interest Statement

The authors declare that the research was conducted in the absence of any commercial or financial relationships that could be construed as a potential conflict of interest.
